# Development and Applications of a Zebrafish (*Danio rerio*) CYP1A-Targeted Monoclonal Antibody (CRC4) with Reactivity across Vertebrate Taxa: Evidence for a Conserved CYP1A Epitope

**DOI:** 10.3390/toxics10070404

**Published:** 2022-07-20

**Authors:** Amy L. Anderson, Benjamin D. Dubanksy, Lindsay B. Wilson, Robyn L. Tanguay, Charles D. Rice

**Affiliations:** 1 Graduate Program in Environmental Toxicology, Department of Biological Sciences, Clemson University, Clemson, SC 29634, USA; amyanderson23@gmail.com; 2 Department of Comparative Biomedical Sciences, Louisiana State University, Baton Rouge, LA 70803, USA; bduban2@lsu.edu; 3 Department of Environmental and Molecular Toxicology, Oregon State University, Corvallis, OR 97333, USA; lindsay.wilson@oregonstate.edu (L.B.W.); robyn.tanguay@oregonstate.edu (R.L.T.)

**Keywords:** zebrafish CYP1A, vertebrate CYP1A, monoclonal antibody (mAb) CRC4, conserved CYP1A epitope, whole mount IHC

## Abstract

CYP1A is a heme-thiolate enzyme associated with the cytochrome P4501A1 monooxygenase system and is inducible by a wide variety of xenobiotics and endogenous ligands that bind and activate the aryl hydrocarbon receptor (AHR). The AHR-CYP1A axis is important for detoxification of certain xenobiotics and for homeostatic balance of endogenous sex hormones, amine hormones, vitamins, fatty acids, and phospholipids. Herein, we generated and described applications of a zebrafish CYP1A-targeted monoclonal antibody (mAb CRC4) that fortuitously recognizes induced CYP1A across vertebrate taxa, including fish, chicken, mouse, rat, and human. We then demonstrated that mAb CRC4 targets a highly conserved epitope signature of vertebrate CYP1A. The unique complimentary determining region (CDR) sequences of heavy and light chains were determined, and these Ig sequences will allow for the expression of recombinant mAb CRC4, thus superseding the need for long-term hybridoma maintenance. This antibody works well for immunohistochemistry (IHC), as well as whole-mounted IHC in zebrafish embryos. Monoclonal antibody CRC4 may be particularly useful for studying the AHR-CYP1A axis in multiple vertebrate species and within the context of Oceans and Human Health research. By using archived samples, when possible, we actively promoted efforts to reduce, replace, and refine studies involving live animals.

## 1. Introduction

The pioneering studies of Professor George Streisinger and colleagues at the University of Oregon using zebrafish to study radiation-induced genetic mutations [1,2,3] eventually led to a highly tractable model for studying vertebrate biology. The subsequent establishment of a specific pathogen-free zebrafish husbandry facility [4,5] paved the way for others to routinely house zebrafish, and ultimately characterize genetic mutations [6]. As the use of zebrafish models continues globally, various mutant strains of zebrafish have contributed to our understanding of fundamental biological processes in nearly all aspects of vertebrate biology, as well as human physiology and disease [7,8,9]. For example, zebrafish are important for understanding vascular and lymphatic development [10,11], brain disorders [12], cancer research [13], and immune functions [14,15].

Within the fields of pharmacology and toxicology, the zebrafish model has significantly contributed to our understanding of the mechanisms of action of xenobiotic drugs and environmental contaminants [16,17]. Zebrafish also provide an excellent platform for drug and toxicant screening [18,19]. Specifically, our current understanding of the aryl hydrocarbon receptor (AHR) and its role in both toxicology and endogenous physiological homeostasis has relied on the zebrafish model for understanding fundamental processes [20,21,22]. The AHR is a member of the basic helix-loop-helix (bHLH-PAS) superfamily of proteins and functions as a ligand-activated transcription factor for a suite of responsive genes [23]. The AHR is highly expressed in barrier epithelial tissues, and thus is an environmental sensor for a variety of AHR ligands [24].

Most notable of the AHR-responsive genes in the fields of toxicology and pharmacology include phase I and II drug-metabolizing enzymes and transporters, as well as the AHR-repressor that functions in a negative feed-back manner to suppress AHR activity [25,26,27,28,29]. Induced expression of CYP1A, a heme-thiolate enzyme associated with the cytochrome P4501A1 monooxygenase system, is an early, yet nonspecific biomarker of AHR activation [28,30]. CYP1A is involved in the metabolism of a wide array of xenobiotic environmental substrates [31] and in recent years it has become clear that the AHR-CYP1A axis is also critical to physiological homeostasis by regulating the metabolism and clearance of endogenous substrates such as sex hormones, amine hormones, vitamins, fatty acids, and phospholipids [32]. This axis is also important for gut homeostasis by regulating concentrations of the endogenous indole ligand 6-Formylindolo [3,2-*b*] carbazole (FICZ) (and others) that in turn regulates the tonic expression of pro-inflammatory vs. anti-inflammatory cytokines [33,34].

In terms of environmental contaminants, adverse effects of exposure to select persistent environmental AHR ligands include immune suppression, reproductive and developmental disorders, as well as metabolic activation of pro-carcinogens and pro-mutagens, and each of these adverse outcomes has CYP1A expression and activity as a key mechanism of action [35,36]. Moreover, as an indicator of AHR activation and activity, the expression of CYP1A protein and its enzymatic activity are routine biomarkers of exposure to environmental AHR ligands in environmental species [37,38]. Likewise, the lack of or reduced CYP1A1 expression in fish during exposure to known environmental AHR ligands is a key indicator of adaptation to contaminants in situ [39,40,41,42,43].

In the study described herein, we generated and described applications of a zebrafish CYP1A-targeted monoclonal antibody (mAb CRC4) that fortuitously recognizes induced CYP1A across vertebrate taxa, including fish, chicken, mouse, rat, and human. We then demonstrate that mAb CRC4 targets a highly conserved epitope signature of CYP1A across vertebrate taxa. This antibody works well in formalin-fixed and paraffin-embedded tissues for immunohistochemistry, as well as whole-mounted zebrafish embryos. Our intention is to make this novel antibody available to the general scientific community, which may be particularly useful for those simultaneously studying the AHR-CYP1A axis in multiple vertebrate species. Monoclonal antibody CRC4 may also be useful for studies within the context of Oceans and Human Health research [44,45,46]. Of importance, by using archived samples, when possible, we actively promoted efforts to reduce, replace, and refine studies involving laboratory animals and wildlife [47].

## 2. Methods

### 2.1. Immunogen Design

A synthetic polypeptide comprising internal amino acids 264-294 zebrafish CYP1A was designed from a full-length zebrafish cDNA sequence (accession # BAB90841.1) and conjugated with keyhole limpet hemocyanin (KLH) (GinoSys-Sigma-Aldrich) for immunizations and as an unconjugated peptide for screening assays. This specific sequence was chosen because several amino acids of the polypeptide differ slightly from other vertebrates and there is an asn/asp difference near the C-terminus (Table 1). The zebrafish peptide also has core sequences shared with other fish and sequences common to all vertebrates.

### 2.2. Monoclonal Antibody Generation

Six-week-old female Balb/c mice (Charles River) were used for immunizations and housed at the Clemson Institute for Environmental Toxicology, Pendleton, SC USA, a Clemson University IACUC approved facility under IACUC-approved protocols. Hybridoma production and screening followed standard methods and procedures from other studies with slight modifications [48]. Mice were given a sub-cutaneous (s.c.) injection with 50 µg in 0.9% saline containing TiterMax Gold^®^ adjuvant on Day 1. Fourteen days later mice received a second s.c. immunization using Freund’s incomplete adjuvant. Subsequent boosters at 21-day intervals were given in saline via s.c. immunizations, and the final booster was given intraperitoneally. Five days after the last booster immunization, mice were sacrificed using slow lethal CO_2_ hypoxia, bled by cardiac puncture, and their spleens removed using aseptic methods. Procedures for fusion of splenocytes with Sp02–14 myelomas (ATCC, Manassas, VA, USA), and for screening and cloning of the resulting hybridomas have been described elsewhere. Hybridomas were typically grown in Dulbecco’s Modified Eagle Medium (Cellgro, Lincoln, NE, USA) supplemented with 10% fetal bovine serum (FBS), 20 mM HEPES, 10 mM L-glutamine, 100 µg/mL penicillin, 100 µg/mL streptomycin, 110 µg/mL sodium pyruvate, 1% non-essential amino acids (from a 100× stock), 4.5 g/L glucose, 10 µg/mL gentamycin, and 5 µg/mL nystatin.

Primary hybridoma supernatants were then screened by ELISA against unconjugated peptide for a minimal signal of three-fold optical density readings above wells probed with an irrelevant antibody (mAb 3F9 against major vault protein) [49], then cloned by limiting dilution for further testing. Hybridomas were grown to confluence and the supernatants collected by centrifugation, then treated with 0.05% NaN_3_ and stored at 4 °C. Initial isotyping of the antibodies in hybridoma supernatants was carried out using Pierce Rapid Antibody Isotyping Kits for mouse (Thermo Fisher). Antibodies were subsequently used as confluent supernatants for most techniques, or further purified if needed. Preliminary studies demonstrated that only three hybridomas secreted antibodies specific to the immunizing peptide. Of these, only one secreted an antibody (mAb CRC4) that recognizes induced zebrafish CYP1A in immunoblotting and other assays, reacts with induced CYP1A in other vertebrates, and recognized the core vertebrate CYP1A sequence IRIDITSLI (produced and supplied by Innovagen AB, Sweden) (further detailed in Results). Immunoglobulin (Ig) genes of the hybridoma expressing mAb CRC4 were then sequenced (Absolute Antibody, Boston, MA, USA) using Next Generation Sequencing (NGS) to verify the presence of a single Ig and of the appropriate isotype. The unique complimentary determining region (CDR) sequences of heavy and light chains were determined, and these Ig sequences will allow for the expression of recombinant mAb CRC4.

### 2.3. Screening mAbs against Cell Line Lysates

To determine the reactivity of mAb CRC4 with zebrafish CYP1A protein, zebrafish liver cells (ZFL) were obtained from ATCC (Manassas, VA, USA) and grown in appropriate media and conditions as directed by the supplier, then transitioned to Leibovitz-15 CO_2_-independent media (L15 media) containing 10% FBS, 4 mM L-glutamine, and antibiotics [50]. Under these conditions the cells grew well for the short duration of the study, but do not grow well over the long term (personal observations by CDR). This medium also supports the growth of frog XLK-WG cells (ATCC, Manassas, VA, USA). Other cells and tissues used for testing and screening are outlined in Table 2. Arochlor 1254-induced and non-induced rat liver S9 fractions were thawed immediately upon receipt from the supplier and prepared for SDS-PAGE-immunoblotting by boiling for 7 min in reducing sample buffer (Thermo Fisher J61337. AD) containing 50 µM PMSF. Commercially sourced HEK cell lysates over-expressing recombinant human CYP1A1 (Origene, Rockville, MD, USA) were prepared similarly.

ZFL, PLHC-1, and HEPA1C1C7 cells were grown to near confluence in 6-well plates (Primavera, Corning). Plates seeded with ZFL cells and PLHC-1 cells were sealed with parafilm and kept in sealed containers to avoid external CO_2_. Cells were treated for 48 h with 10 µM PCB-126 (CAS 57465-28-8, Ultra Scientific) dissolved in DMSO or 0.01% DMSO as a carrier control. XLK-WG cells were grown to near confluence in sealed T-75 flasks, then treated for 12 h with 50 nM of the AHR ligand 6-Formylindolo [3,2-*b*] carbazole (FICZ) (CAS 172922-91-7, Tocris Biosciences, Bristol, UK) in DMSO or 0.01% DMSO, and again for an additional 12 h with the same. HEK cells were grown in media only as a cell-specific source of control cell lysate for comparison with over-expressed recombinant CYP1A1 in HEK cell lysates from the supplier.

Media was then removed from cells, replaced with 0.25% trypsin, and incubated until cells lifted from attachment. Cells were then pelleted by centrifugation to obtain a dry pellet, covered with ice-cold 300–400 µL RIPA buffer (Thermo Fisher, Waltham, MA, USA) supplemented with 2× HALT protease inhibitor cocktail (Pierce), followed by resuspension using a vortex device and placement on ice for 20 min. Suspensions were then sonicated, transferred to 1.5 mL tubes, and centrifuged 12,000× *g* for 20 min. Overlying supernatants were removed and transferred to clean tubes and the protein content quantified. Lysate protein concentration for each cell line, rat S9 fractions, and rainbow trout microsomes were matched between treated and controls, then boiled in reducing sample buffer for 7 min. Forty micrograms of each sample was then subjected to SDS-PAGE using 10-lane, 50 µL well capacity 4–20% pre-cast gels (Biorad, Richmond, CA, USA) and immunoblotting using standard procedures incorporating PDVF membranes. Immunoblots were blocked overnight with 10% horse serum (Gibco) in 0.01 M phosphate buffered saline (PBS) containing 0.05% tween twenty (PBS-T). Membranes were washed once for 10 min with PBS-T, then probed for 2 h at room temperature (RT) with mAbs as confluent supernatants diluted 1:10 in PBS-T containing a final nominal concentration of 3 ug/mL antibody [54]. The membranes were washed 3× fr 10 min with PBS-T, then incubated for 2 h with goat-anti-mouse IgG conjugated with alkaline phosphatase (AP) (Invitrogen #31320) (1:2000 in PBS-T). After 3× washes with PBS-T, membranes were incubated with NBT-BCIP (Fisher Scientific, Pittsburgh, PA, USA) as the AP substrate and activity was visualized as dark blue banding of CYP1A at the expected size of approximately 55 kDa. Blots were dried at room temperature and documented using a ChemiDoc™ imaging system (Biorad) to compare samples from each represented vertebrate.

### 2.4. Whole Mount Embryo Immunohistochemistry

Specific pathogen-free 5D zebrafish were reared at the Sinnhuber Aquatic Research Laboratory (SARL) in accordance with Institutional Animal Care and Use Committee protocols at Oregon State University (IACUC-2021-0166 and 2021-0227). Fish were housed on a recirculating water system kept at 28 ± 1 °C under a 14 h light: 10 h dark cycle in brood stock tanks (50 or 100 gallon). Water was supplemented with Instant Ocean salts (Spectrum Brands, Blacksburg, VA, USA) and sodium bicarbonate to maintain pH 7.4. Fish were fed twice daily with Gemma Micro (Skretting, Inc., Fontaine Les Vervins, France).

Embryos were collected on the day of exposure, sorted by developmental stage, and kept in E2 embryo medium (15 mM NaCl, 0.5 mM KCl, 1 mM CaCl_2_, 1 mM MgSO_4_, 0.15 mM KH_2_PO_4_, 0.05 mM Na_2_HPO_4_, and 0.7 mM NaHCO_3_ buffered with 1 M NaOH to pH 7.2), hereafter referred to as EM, in a temperature-controlled incubator at 28 ± 1 °C until dechorionation. At 4 h post-fertilization (hpf), embryos were dechorionated enzymatically using a custom-made apparatus [55]. Following dechorionation, embryos were screened for enzymatic or mechanical damage under a dissecting microscope and robotically loaded into 96-well plates pre-filled with 100 µL EM.

CYP1A expression was induced in vivo by exposure to retene (CAS 483-65-8, Santa Cruz Biotechnology, 97% purity), a potent AHR activator [56,57]. Retene and 100% dry dimethyl sulfoxide (DMSO control) were dispensed directly into 96-well plates pre-loaded with 6 hpf embryos and 100 µL EM using an D300e Digital Dispenser to achieve 0 or 30 µM retene concentration normalized to 0.1% DMSO. Plates were immediately sealed with pressure-sensitive silicone adhesive backed polyolefin plastic PCR film (ThermalSeal RTS, Excel Scientific) and incubated overnight at 28 ± 1 °C in the dark on an orbital shaker at 235 RPM.

At 24 hpf, the plates were removed from the shaker and incubated in the dark at 28 °C until 48 hpf. At 48 hpf, embryos were transferred to 1.5 mL microcentrifuge tubes and anesthetized on ice in groups of 10. Embryos were euthanized with buffered Tricaine^®^ and the solution was immediately removed and replaced with 500 µL 4% paraformaldehyde in PBS. Tubes were kept on a rocker overnight at 4 °C to fix tissues. The following morning, fish were rinsed 3 times with PBS then stored in 0.02% NaN_3_ in PBS (PBS-NaN_3_) preservative at 4 °C until immunohistochemistry was performed.

For whole-mount-IHC, fish were transported to mesh-bottom well plate inserts in a 24-well plate and the solution was removed. Fish were then washed 3 times for 15 min each in PBS with 0.1% Tween^®^ 20 detergent (PBST). For each wash step throughout the procedure, fish were kept in solution with gentle manual agitation for the designated duration. Well inserts containing fish were moved to a new well with fresh solution for the subsequent step.

Fish tissue was permeabilized using freshly made 0.005% trypsin in PBS for 8 min on ice. Following permeabilization, fish were washed 3 times for 5 min each in PBST then post-fixed with 4% PFA for 10 min at room temperature and washed again twice for 5 min with PBST. Ten percent normal goat serum in PBST with 0.01% triton X-100 (PBSTx) was used to block non-specific protein binding for 10 min at room temperature. Following the blocking step, fish were transferred to 1.5 mL microcentrifuge tubes and NGS-PBSTx solutions were removed. Primary antibody (mAb CRC4) as confluent hybridoma supernatant was diluted 1:10 in NGS-PBSTx to yield a nominal concentration of 3 µg/mL and added to tubes and incubated overnight at 4 °C.

After overnight incubation, the primary antibody was removed, and fish were transferred to well plate inserts and rinsed in PBST twice for 5 min and 4 times for 30 min each. Fish were then incubated with goat anti-mouse IgG1-Alexa Fluor 594 (1:1000, Invitrogen A-21125) secondary antibody for 2 h at room temperature. The secondary antibody was removed, and fish were rinsed in PBST twice for 5 min and 4 times for 30 min each then stored in PBS-NaN_3_ until imaging. To assess CYP1A protein localization, fish were imaged in a 12-well glass bottom plate using a Keyence BZ-X710 fluorescence microscope (Keyence, Osaka, Japan) using a Texas Red filter cube. Representative fish from each group were mounted on 35 mm glass bottom dishes (Matsunami Glass, Bellingham, WA, USA) and imaged in the same manner. Images were then converted to 32-bit gray scale and recorded for demonstration of CYP1A expression.

### 2.5. Tissue Immunohistochemistry

Archived tissues used in previous studies examining AHR ligand activation were re-analyzed using mAb CRC4 (Table 2). Grossly dissected archived tissues (fixed and stored at 4 °C in 70% ethanol) were processed and embedded in ParaPlast Extra by the Louisiana Animal Disease Diagnostic Laboratory (LADDL) at Louisiana State University School of Veterinary Medicine. Newly embedded tissues were then sectioned and affixed to charged slides for immunohistochemistry. 

Mouse liver tissues used for this study were from previous studies wherein mice were exposed to 1 mg/kg PCB-126 prior to tissue fixation [51]. Archived rainbow trout livers (and microsomes stored at −80 °C from beta-naphthoflavone (β-NF)-treated animals from a previous study [48] were used for immunohistochemistry and immunoblotting (described above), respectively). Archived liver tissue from Gulf killifish collected from sites affected by the Deepwater Horizon spill (DHOS) [58,59,60] were analyzed for CYP1A alongside livers from a lab-held colony of CYP1A-recalcitrant Gulf killifish, which is used as a molecular control (naturally acquired CYP1A knockdown) [60]. Finally, previously analyzed embryonic chicken livers exposed to airborne volatiles from DHOS oil were included for additional species comparison [52].

Zebrafish (strain AB) exposed to PCB-126 (Ulra Scientific) were also used for this study. Zebrafish were housed at the former Clemson Institute of Environmental Toxicology, Pendleton SC and maintained under Clemson University IACUC approved methods. Two juvenile male and two juvenile female zebrafish (40 dpf) were exposed for 48 h to either 10 µM PCB-126 or 0.10% DMSO in 1 L glass bottles containing maintenance water from the recirculating system and supplied with gentle aeration. Following exposure, the 4 fish per treatment were euthanized on ice with buffered Tricaine^®^, their abdominal cavities opened, and placed in in 10% aqueous buffered zinc formalin (Z-Fix, Anatech, Ltd., Battle Creek, MI, USA) for 2 days, and the fixative replaced with 70% ethanol and stored at 4 °C. Prior to processing, zebrafish were decalcified in 0.35 M EDTA, pH 7.8 at room temperature (RT) for 6 days [61], then processed for microtomy as above, at LADDL. Fish and chicken tissue IHC was performed using a horse-anti-mouse IgG VectaLabs ABC-Elite kit (PK-6102), while mouse tissue IHC was performed using the mouse-on-mouse (M.O.M) anti-mouse IgG VectaLabs ABC-Ultra kit (BMK-2202.)

Whole zebrafish tissues were sectioned at 7–9 μm and mouse, trout, killifish, and chicken tissues were sectioned at 4–5 μm on a Microm 325 rotary microtome. Prior to antibody labeling, antigen retrieval studies were carried out to determine the optimal conditions for mAb CRC4 binding using zebrafish tissues. A moderate heat cycle with an alkaline pH was determined to be ideal. Subsequently, slides were immersed in pH 9 Tris-EDTA buffer while heating by microwave on 100% power for 5 min followed by cooling for 5 min, followed by a final 5 min 100% power, then a final 20 min cool-down period in the container at room temperature. After antigen retrieval, tissues on slides were encircled with a Liquid Blocker Super mini pen to isolate tissue slices. Thus, each slide was divided into sections where one section can act as a reagent control by withholding a primary antibody. Additional screening assays included isotype controls. Once slides were divided, all sections were treated to quench endogenous peroxidase activity for 30 min using 3% H_2_O_2_ in water. Following quenching, a 30 min blocking step was conducted using PBS, pH 7.4 with horse serum provided in the horse-anti-mouse IgG VectaLabs ABC-Elite kit (PK-6102) plus 1% fetal bovine serum (FBS.) After blocking, avidin- then biotin-blocking steps (15 min each) were conducted to allow specificity to target molecules only for the avidin–biotin binding. Next, the antibody, as a hybridoma supernatant diluted 1:10 in PBS with 0.5% FBS, was added dropwise to the slides and incubated overnight at 4 °C or for 2 h at RT. After incubation, the slides were washed prior to addition of the secondary (biotinylated) antibody for a 30 min incubation at RT. After washing, Vectastain ABC reagent was added for 30 min prior to staining. Following the manufacturer’s instructions, ImmPACT Nova Red (SK-4805) was then used to visualize mAb CRC4 distribution. Since mAb CRC4 is a mouse antibody, the M.O.M anti-mouse IgG Vectalabs ABC-Ultra kit (BMK-2202) was used to detect CYP distribution in mouse tissues. Mouse tissue IHC followed the same general protocol for probing and visualization, but with some slight changes directed by the M.O.M. kit (VectaLabs). To complement ImmPact^®^Nova Red^®^ staining for CYP1A, tissues were counter-stained with hematoxylin Q.S. (Vector Labs).

Stained slides were imaged using a NanoZoomer 2.0-HT slide scanner (Hamamatsu). Slide areas were scanned at 40× resolution and images from scans were exported into TIFF format. All liver tissue images were adjusted for levels in Adobe Photoshop, with RAW images preserved in native format. Histogram normalization of micrographs was used to enhance contrast.

## 3. Results

### 3.1. Generation of Monoclonal Antibodies

**Figure 1:**
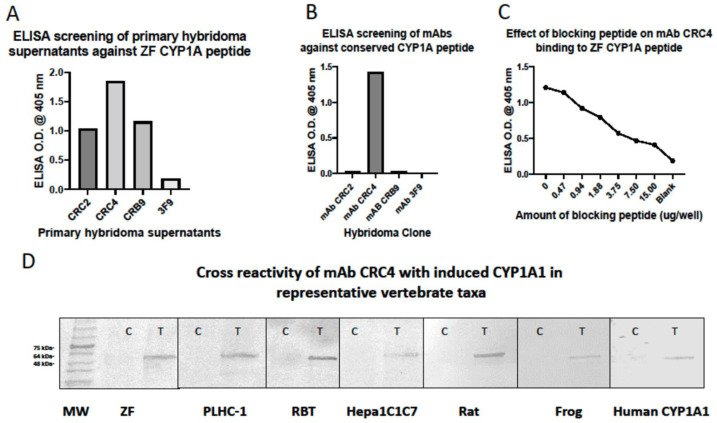
(**A**). ELISA results demonstrating reactivity of three primary hybridoma supernatants against zebrafish CYP1A peptide used for immunizations. (**B**). ELISA results demonstrating that only mAb CRC4 recognizes a conserved peptide sequence common to all vertebrates. (**C**). ELISA results demonstrating that the conserved CYP1A sequence blocks the binding of mAb CRC4 to ELISA plate-bound peptide used for immunizations. (**D**). Cross reactivity of mAb CRC4 with induced CYP1A protein in representative vertebrates. MW, molecular weight markers; C (control); T (treated); ZF, zebrafish liver cells; PLHC-1, *Poeciliopsis lucida* hepatoma cell line; RBT, β-NF induced rainbow trout microsomes; HEPA1C1C7, mouse hepatoma cell line; Rat, liver S9 fractions; frog, XLK-WG cell line; human CYP1A1, recombinant CYP1A1 expressed in HEK cells. Details are found in Methods and Table 2.

Three hybridomas secreting monoclonal antibodies (mAb) meeting selection criteria were developed and characterized as mAb CRC2 (IgG_2a_ κ), mAb CRC4 (IgG_1_ κ), and mAb CRC9 (IgG_1_ κ) (Figure 1A). Subsequent studies demonstrated that only mAb CRC4 recognized induced zebrafish CYP1A protein by immunoblotting. Using additional screening approaches, ELISAs were carried out to determine if either of the three mAbs would recognize the core vertebrate CYP1A sequence IRIDITSLI (produced and supplied by Innovagen AB, Sweden). Only mAb CRC4 recognized this peptide sequence by ELISA (Figure 1B). An additional ELISA system was used to determine if this conserved peptide could be used to block the binding of mAb CRC4 to the zebrafish CYP1A peptide used for immunizations. Based on the general rule of thumb that confluent supernatants contain approximately 30 µg/mL of specific antibody [54], a total of 60 µg of the peptide in 100 µL PBS as the highest concentration was serially diluted and 50 µL of each dilution were mixed with 50 µL confluent supernatants to yield 100 µL of total mixture for screening ELISA plates coated with the original zebrafish peptide. This mixture of hybridoma supernatant and peptide dilutions was allowed to incubate overnight at 4 °C. The next day these mixtures of 100 µL total sample were mixed by gentle agitation and added to zebrafish CYP1A1 peptide-pre-coated ELISA plates and incubated for 2 h at RT. After extensive washings with PBST, the plates were incubated with goat anti-mouse IgG conjugated with alkaline phosphatase for 2 h, washed extensively, and developed and quantified at 405 nm and the data recorded. The estimated ratio of 1:1 conserved peptide and antibody (15 µg peptide:15 µg antibody) resulted in the most inhibition of activity (Figure 1C), thus providing additional evidence that this sequence contains the epitope recognized by the antibody.

### 3.2. Cross Reactivity of mAb CRC4 against Induced CYP1A1 across Vertebrate Taxa

Treatment (T)-induced CYP1A protein was recognized by mAb CRC4 across all vertebrate taxa examined (Figure 1D). No CYP1A protein was detected in either of the controls (C).

### 3.3. Detecting Induced Embryonic Expression of Zebrafish CYP1A Using mAb CRC4

**Figure 2:**
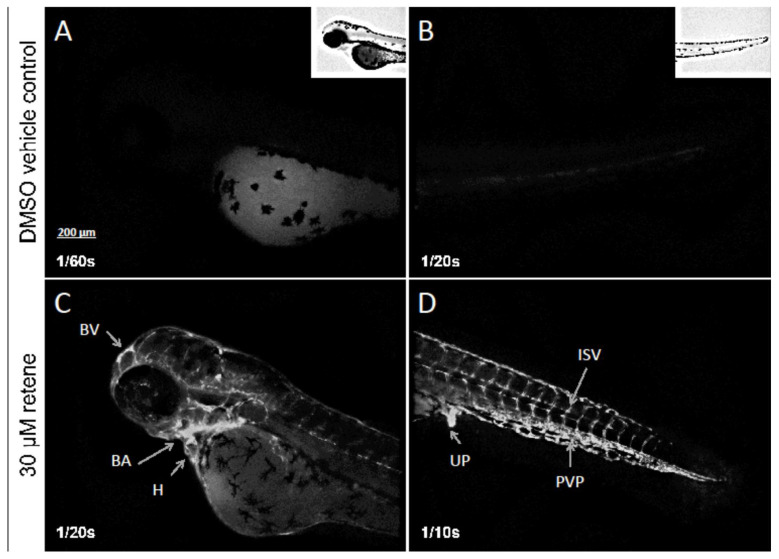
Whole-mount immunolocalization of zebrafish CYP1A protein in a representative DMSO vehicle (control) larva (**A**,**B**) and 30 µM retene-exposed (**C**,**D**) larvae at 48 hpf using mAb CRC2. Each larva was exposed to their respective treatment from 4 hpf through 48 hpf. BA, brachial vasculature; BV, brain vasculature; H, heart; UP, urogenital pore; PVP, posterior vascular plexus; ISV, intersegmental vessel. Details are found in Methods.

Using detailed methods for whole-embryo immunohistochemistry, we show that mAb CRC4 detects retene-induced CYP1A expression in the vasculature throughout the embryo, with intense staining near the heart and branchial artery, but also in the tail region and urogenital pore (Figure 2).

### 3.4. Detecting Induced CYP1A Expression in Juvenle Zebrafish Tissues Using mAb CRC4

**Figure 3:**
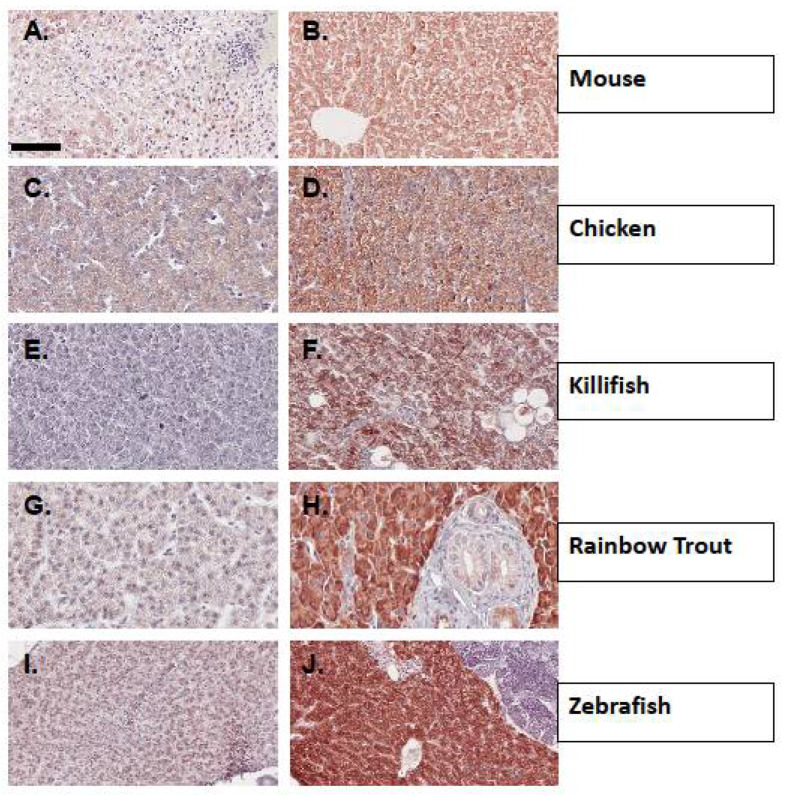
Immunohistochemical detection of CYP1A protein expression using mAb CRC4 in liver tissues from representative vertebrates. (**A**). Control mouse liver; (**B**). Liver from a PCB-126 treated mouse; (**C**). Control chicken embryo liver at 18 days post fertilization (DPF); (**D**). Liver from a crude oil vapor-exposed chicken embryo at 18 DPF; (**E**). Liver from CYP1A recalcitrant Gulf killifish; (**F**). Liver from Gulf killifish collected from a crude oil exposed site; (**G**). Control Rainbow trout liver; (**H**). Liver from a β-NF-exposed rainbow trout; (**I**). Control zebrafish liver; (**J**). Liver from PCB-126 treated zebrafish. (**A**,**B**) are 20× magnification, scale bar = 100 µm (**C**–**J**) are 40× magnification, scale bar = 50 µm. Details of tissue sources, experimental conditions, and procedures are found in Methods and Table 2.

Using standard immunohistochemistry protocols, we show that CYP1A is easily detected in PCB-126-exposed mouse livers but not unexposed controls (Figure 3A,B), as well as chicken embryo livers exposed to vapors from crude oil 18 days post fertilization (DPF) (Figure 3C,D). Likewise, crude oil-induced CYP1A expression is easily detected in adult gulf killifish but not in individuals from a genetically resistant population located within the upper Houston Ship Channel (Figure 3E,F). Some of the most intense staining of CYP1A by mAb CRC4 was found in rainbow trout livers treated with β-NF just prior to tissue removal and fixation (Figure 3G,H) and in zebrafish adult livers from animals exposed to PCB-126 (Figure 3I,J) prior to tissue removal and fixation.

## 4. Discussion

In this study we generated zebrafish CYP1A-specific mAbs that were initially intended for zebrafish-specific research. Subsequent characterization revealed that one antibody (mAb CRC4) recognized induced CYP1A not only in zebrafish, but all vertebrates examined, and that the epitope for the antibody is the highly conserved vertebrate peptide IRTIDSLI. The timing of our study may be fortuitous considering the limited availability of other commonly used CYP1A-specific antibodies. Nearly 40 years ago, mAb 1-12-3 was generated against Scup, *Stenotomus chrysops*, CYP1A and shown to recognize CYP1A in rats as well [62,63]. Thanks to the generosity of Dr. John Stegeman (Woods Hole Institutions of Oceanography), mAb 1-12-3 has been used in numerous studies over the past decades and has contributed greatly to basic toxicology and environmental toxicology in general. Those studies and others clearly showed that mAb 1-12-3 recognizes CYP1A in all vertebrates [64]. Unfortunately, hybridoma 1-12-3 has been lost and is no longer available (personal communications with Dr. Stegeman). Almost 30 years ago, mAb C10-7 was generated against rainbow trout CYP1A and shown to be cross reactive with all fish examined [48], and since then, mAb C10-7 has been available commercially as “anti-fish CYP1A” from several sources, including Biosense and Cayman. To date, we (CDR) have not been able to demonstrate mammalian CYP1A detection by mAb C10-7. Unfortunately, hybridoma C10-7 has also been lost at the originator’s (CDR) current institution, thus stressing the importance of having other CYP1A-specific mAbs available to the scientific community, and especially the ability to express recombinant forms of the antibody that supersede the need to maintain hybridomas long term. To this end, the unique complementary determining regions (epitope binding regions) of heavy and light chains of hybridoma CRC4 have been sequenced, which will allow us to produce a recombinant mAb CRC4.

We demonstrated that mAb CRC4 recognizes induced CYP1A across vertebrate taxa and based on the conserved core sequence recognized by the antibody, we predict that induced reptilian CYP1A will be recognized as well. Of note, the staining intensity of induced frog CYP1A in immunoblots was less than for the other samples, which may be reflective of the low affinity of amphibian AHR for ligands [65,66]. Nonetheless, mAb CRC4 should be useful for understanding the AHR-CYP1A axis in amphibians. As we demonstrate, whole-mount zebrafish embryo CYP1A induction by the AHR ligand retene is easily detected by mAb CRC4, with the vasculature being a sensitive target. Many environmental AHR ligands are associated with embryo toxicity that involves vascular malformation, deformed heart development, and the general phenomenon of blue sac’s disease (BSD); these adverse outcomes correlate with CYP1A induction in the vascular endothelium [67]. In general, it appears that vascular endothelium is a sensitive target for strong AHR ligands regardless of taxa [68,69]. As a model species, zebrafish vasculature development is well characterized, and the reader is referred to other sources for the fine details of which vessels are stained in our whole-mount figure [70].

As with endothelial cells, hepatocytes (and bile duct epithelium), which form a barrier epithelium within the liver, are sensitive to AHR ligands and highly express CYP1A, as shown in our liver IHC figures using mAb CRC4. The differences in staining intensity between mouse, chicken, and fish livers by the antibody may be due to antigen retrieval details. Preliminary studies using zebrafish tissues indicated that antigen retrieval using microwave heating and Tris-EDTA, pH 9 conditions resulted in more favorable staining when compared to low pH conditions (citrate buffer, pH 6), or without antigen retrieval. Therefore, all tissues were subsequently probed using heat and high pH. Yet, it is possible that other conditions would improve staining in species other than fish, and future studies should examine alternatives. Additional caution is needed when probing whole-mouse tissues with a mouse antibody because of the high background of IgG that would be recognized by secondary antibodies. However, by using mouse-on-mouse reagents, such as VectaLabs M.O.M. kits, this can be overcome for the most part. How or if this constraint affects staining intensity in whole-mouse tissues is not yet clear. Staining of chicken tissues with mAb CRC4 yields the same intensity as seen using mAb C10-7 [52].

In summary, the development of mAb CRC4 adds to the growing toolbox of reagents that can be used for AHR-CYP1A axis research. For example, this antibody could be routinely used in studies involving drug-toxicant screening, to validate knock down or knock out of key components of AHR-CYP1A axis, as a tool for ecotoxicologists to detect exposure to environmental AHR ligands, and to immune-purify CYP1A from a variety of vertebrates that could in turn be used to generate other antibodies for use in capture ELISA assays. Within the near future, we will have recombinant mAb CRC4 expressed in HEK or CHO cells that then supersedes the need to maintain the hybridoma along term.

## Figures and Tables

**Table 1:** Amino acid sequence of predicted most immunogenic CYP1A peptides (underlined) from representative vertebrates (F, fish; Rp, reptile; Avian, Av; Mammal, M) demonstrating a highly conserved core CYP1A1 epitope (IRDITDSLI). Mice were immunized with synthetic zebrafish CYP1A peptide conjugated to KLH at the C-terminus cysteine (C) and resulting hybridomas screened against the unconjugated peptide by ELISA. Positive hybridomas were subsequently re-screened by ELISA against the core sequence (IRDITDSLI) to obtain mAb CRC4 with reactivity across vertebrate taxa.

CYP1A Amino Acid Sequence	Species	Accession Number
VMEHYDTFDKD**NIRDITDSLIN**HC	Zebrafish, *D. rerio (F)*	BAB90841.1
VSEHYESYDKD**NIRDITDSLI**DHC	Rainbow trout, *O. mykiss (F)*	AAB69383.1
VSDHYDTFDKD**NIRDITDSLI**DHC	Fathead minnow, *P. promelas (F)*	XP_039536214.1
VSEHYTTFDKD**NIRDITDSLI**DHC	Scup, *S. chrysops (F)*	AAA74969.1
VSEHYSTFDKD**NIRDITDSLI**DHC	Mummichog, *F. heteroclitus (F)*	AAD01809.1
VTEHYHTFDKD**NIRDITDSLI**DHC	Whale shark, *R. typus (F)*	XP_020387799.1
VREHYDTYDKD**NIRDITDSLI**DHC	Channel catfish, *I. punctatus (F)*	XP_017321977.1
VKEHYSSFDKD**NIRDITDSLI**EHC	Green Sea turtle, *C. midas (Rp)*	EMP30185.1
VEEHYQTFDKN**NIRDVTDSLI**EQC	Chicken, *G. gallus (Av)*	NP_990477.2
TKEHYKTFDKN**HIRDITDSLI**QHC	African clawed frog, X. *laevis (Am)*	NP_001090813.1
VKEHYKTFDKS**HIRDITDSLI**EHC	American alligator, *A. miss. (Rp)*	KYO21524.1
IKEHYRTFEKG**HIRDITDSLI**EHC	Mouse, *M. musculus (M)*	NP_001129531.1
IKEHYRTFEKG**HIRDITDSLI**EHC	Rat, *R. norvegicus (M)*	NP_036672.3
VKEHYKTFEKG**HIRDITDSLI**EHC	Human, *H. sapiens (M)*	P04798.1
**IRDITDSLI**	Conserved CYP1A1 epitope	All the above

**Table 2:** Cells and tissues used in this study. Cells were grown and maintained as directed from the source or as modified (see Methods). Tissues from laboratory or environmentally exposed animals were processed for either tissue sub fractionation, formalin fixation and paraffin embedding followed by immunohistochemistry (IHC), or whole-mount IHC as described in the Methods section. Referenced sources were archived samples from previous studies (see Methods).

Animal Cell Lines and Tissues	Origin	Source
ZFL	Zebrafish liver cells	ATCC CRL-2643
PLHC-1	Top minnow hepatocellular carcinoma	ATCC CRL-2406
Hepa1c1c7	Mouse liver hepatoma	ATCC CRL-2026
XLK-WG	African clawed frog kidney epithelial	ATCC CRL-2527
HEK293	Human embryonic kidney	ATCC CRL-3467
Rat liver S9 fraction	Arochlor-1254-induced P450s	MOLTOX 11101.5
Rat liver S9 fraction	Non-induced livers	MOLTOX 11102.2
Recombinant human CYP1A	Over-expressed in HEK293 cell lysates	OriGene LC400170
Mouse livers for IHC	PCB-126-induced CYP1A (and controls)	[51]
Rainbow trout liver microsomes & tissue	Β-NF-induced CYP1A (and controls)	[48]
Chicken embryo liver	Crude oil induced CYP1A (and controls)	[52]
Adult zebrafish for IHC	PCB-126 induced CYP1A (and controls)	This study (see Methods)
Larval zebrafish for whole mount IHC	Retene-induced CYP1A (and controls)	This study (see Methods)
Gulf killifish for IHC	Tissues from Deep Water Horizon studies	[53]

## Data Availability

Data are contained within the article.
